# Identification of Novel Pepper Genes Involved in Bax- or INF1-Mediated Cell Death Responses by High-Throughput Virus-Induced Gene Silencing

**DOI:** 10.3390/ijms141122782

**Published:** 2013-11-19

**Authors:** Jeong Hee Lee, Young Cheol Kim, Doil Choi, Jeong Mee Park

**Affiliations:** 1 Infection and Immunity Research Center, Korea Research Institute of Bioscience & Biotechnology (KRIBB), 125 Gwahak-ro, Yusung-gu, Daejeon 305-600, Korea; E-Mails: jhlee@seeders.co.kr (J.H.L.); mbgenes@naver.com (Y.C.K.); doil@snu.ac.kr (D.C.); 2 Department of Plant Science, Seoul National University, Seoul 151-921, Korea

**Keywords:** Bax-induced cell death, hot chili pepper (*Capsicum annum*), hypersensitive response, INF1, *Nicotiana benthamiana*, virus-induced gene silencing

## Abstract

Hot pepper is one of the economically important crops in Asia. A large number of gene sequences, including expressed sequence tag (EST) and genomic sequences are publicly available. However, it is still a daunting task to determine gene function due to difficulties in genetic modification of a pepper plants. Here, we show the application of the virus-induced gene silencing (VIGS) repression for the study of 459 pepper ESTs selected as non-host pathogen-induced cell death responsive genes from pepper microarray experiments in *Nicotiana benthamiana*. Developmental abnormalities in *N. benthamiana* plants are observed in the 32 (7%) pepper ESTs-silenced plants. Aberrant morphological phenotypes largely comprised of three groups: stunted, abnormal leaf, and dead. In addition, by employing the combination of VIGS and *Agrobacterium*-mediated transient assays, we identified novel pepper ESTs that involved in Bax or INF1-mediated cell death responses. Silencing of seven pepper ESTs homologs suppressed Bax or INF1-induced cell death, five of which suppressed both cell death responses in *N. benthamiana*. The genes represented by these five ESTs encode putative proteins with functions in endoplasmic reticulum (ER) stress and lipid signaling. The genes represented by the other two pepper ESTs showing only Bax-mediated cell death inhibition encode a CCCH-type zinc finger protein containing an ankyrin-repeat domain and a probable calcium-binding protein, CML30-like. Taken together, we effectively isolated novel pepper clones that are involved in hypersensitive response (HR)-like cell death using VIGS, and identified silenced clones that have different responses to Bax and INF1 exposure, indicating separate signaling pathways for Bax- and INF1-mediated cell death.

## 1. Introduction

Many plant DNA sequences, including whole genome sequences and expressed sequence tags (EST), have been released in the last decade. Developments in EST and cDNA microarray technology have allowed the responses of thousands of genes associated with intercellular and intracellular signaling pathways to be investigated. However, although this large body of information has accelerated the characterization of responses at the genomic level, the ability to functionally investigate individual genes remains limited. Some economically important crops, such as pepper, are particularly intractable for reasons such as reduced susceptibility to genetic transformation.

Generation of gain- and loss-of-function mutations is a common strategy to investigate gene function. In particular, in some plant species, knock-out mutant populations can be produced by using T-DNA insertions or site-directed mutagenesis; however, embryonic lethal mutations and redundant genes can be overlooked using this strategy. Virus-induced gene silencing (VIGS) is an alternative genetic tool that allows functional characterization by knocking-down the expression of native genes [1,2]. VIGS is particularly useful when applied to genes in which mutations are embryonic lethal and in species that are resistant to transformation. In addition, VIGS has been adapted for high-throughput functional genomics [3,4] and, by using heterologous sequences, has been successfully used for species that lack genomic sequence availability [5]. Both pepper and *N. benthamiana* are within the Solanaceae family and it has been approved in several publications that functional characterization of pepper genes in *N. benthamiana* is a useful using tobacco rattle virus (TRV)-mediated gene silencing methods [6–8].

Programmed cell death that occurs during plant-pathogen interactions is well characterized [9,10]. During incompatible plant-pathogen interactions, a rapid and localized cell death is triggered at the infection site, termed the hypersensitive response (HR); this is similar to apoptosis in animals. This HR is often associated with resistance to further pathogen multiplication and spread [9,10]. Although details of the molecular signal pathway of HR cell death remain poorly understood, production of reactive oxygen species (ROS), and gene activation have been elucidated. Plant homologs of animal apoptotic regulators such as the Bcl-2 family proteins have been identified [11,12]. Bax is a member of the Bcl-2 family of proteins that contains crucial managers of cell survival and cell death. Several reports indicate that the control and execution of programmed cell death in animal and plant cells is evolutionarily conserved. For example, transient overexpression of Bax using a viral vector induces cell death in tobacco [11], and this cell death response induced ROS production and pathogenesis-related gene expression, like HR mediated by tobacco *N* gene [11]. In addition, overexpression of *Arabidopsis* AtBI-1, a plant homolog of the mammalian Bax inhibitor, inhibits Bax-induced cell death in *Arabidopsis* as well as fungal elicitor-induced cell death in cultured rice cells [12,13]. INF1 is a 10 kDa extracellular protein produced by the oomycete *Phytophthora infestans*, the causal agent of late blight disease in potato and tomato [14]. INF1 triggers a wide range of defense responses, including cell death, in most *Nicotiana* species [15]. Signaling cascades following INF1 recognition, which lead to hypersensitive cell death, have been studied [16–18]. Thus far, the very limited numbers of host proteins required for Bax- or INF1-mediated cell death have been determined through transient expression study in *N. benthamiana* and *Arabidopsis* mutant analyses. To study the function of pepper ESTs that specifically regulated during pepper and non-host pathogen interaction, we employed the TRV-mediated VIGS approach. Genes were silenced in *N. benthamiana* plants using VIGS with 459 EST clones and the morphological phenotypes of the plants (7%) were observed. Cell death was further investigated by the transient expression of the cell death inducers Bax and INF1 in leaves of silenced *N. benthamiana* plants. To date, seven pepper genes, including catalytic activity-related, binding activity-coupled, and transcription factor genes, were found to be involved in Bax- and INF1-induced cell death.

## 2. Results and Discussion

### 2.1. Silencing of Pathogen-Induced Pepper ESTs in *N. benthamiana* Plants

**Figure 1:**
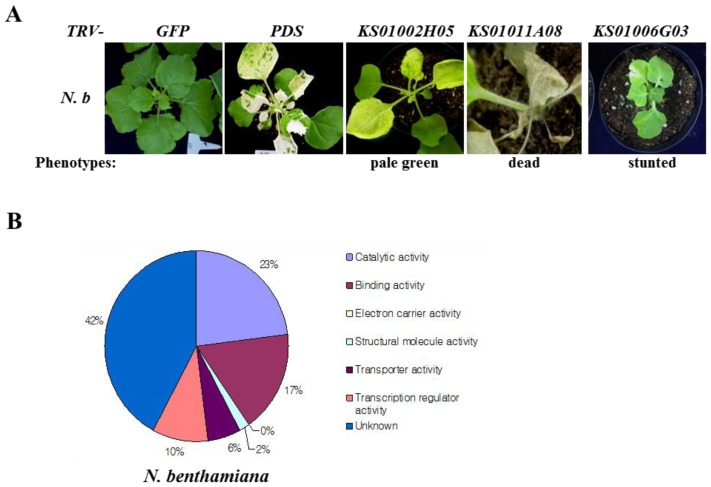
Morphological phenotypes in VIGSed *N benthamiana* plants. (**A**) Representative morphological phenotypes in *N. benthamiana* after VIGS. GFP negative and PDS positive controls for the VIGS experiment, respectively; *KS01002H05*, *KS01011A08*, *KS01006G03*, characteristic aberrant phenotypes of the classes pale green, dead, and stunted, respectively. Images of the silenced plants were taken 21 days post-infiltration, respectively; and (**B**) Molecular characterization of pepper ESTs exhibiting morphological abnormalities. Percentages were calculated using the total numbers in *N. benthamiana*.

Previously, we performed hot chili pepper cDNA microarray analysis with 4685 unique EST clones, and 613 ESTs that were differentially expressed when exposed to the soybean pustule pathogen *Xanthomonas axonophodis* pv. *glycines* 8ra (*Xag* 8ra) were isolated [19]. *Xag* 8ra is not a pathogen of pepper, but does elicit an HR-like cell death in pepper leaves, as well as induce the expression of a number of pathogenesis-related (PR) genes [19]. For VIGS analysis, 459 of these ESTs were successfully ligated into the *pTRV2* vector. Details of the *pTRV2* derivatives are provided in Supplementary File 1. Plants infected with *TRV-PDS* or *TRV-GFP* were used as positive and negative controls for observation of the morphological change caused by VIGS experiment, respectively [20,21]. VIGS experiments were performed with two-week-old *N. benthamiana* plants using previously described methods [21]. At three weeks after co-infiltration of *N. benthamiana* plants with *pTRV1* and *pTRV2* constructs, morphological abnormalities such as chlorosis, leaf deformations, and stunted growth were compared with that of *GFP*-silenced control plants. Using data from two biologically independent VIGS experiments, plants were categorized based on their phenotypes, with most plants falling into one or more of four main groups: normal, stunted, abnormal leaf, and dead (Table 1; Figure 1A). Plants were silenced with constructs derived from 459 pepper ESTs. Of these, 32 *N. benthamiana* plants (7%) exhibited a different morphology from that of the corresponding *GFP*-silenced control plants. The low proportion of the non-host pathogen-induced genes that exhibited abnormal growth under normal growth condition in the silenced plants is consistent with their specific induction by a non-host pathogen. The pepper genes that exhibited morphological differences when silenced were categorized by molecular function (Figure 1B).

### 2.2. Screening of Pepper Genes Coupled with Bax- or INF1-Induced Cell Death Responses

**Figure 2:**
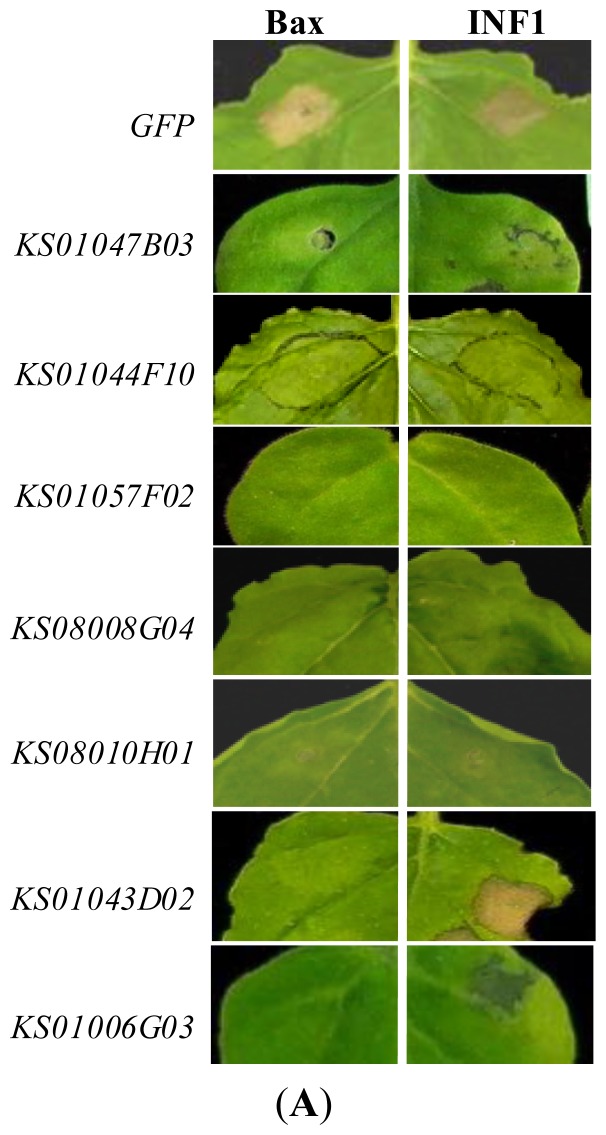

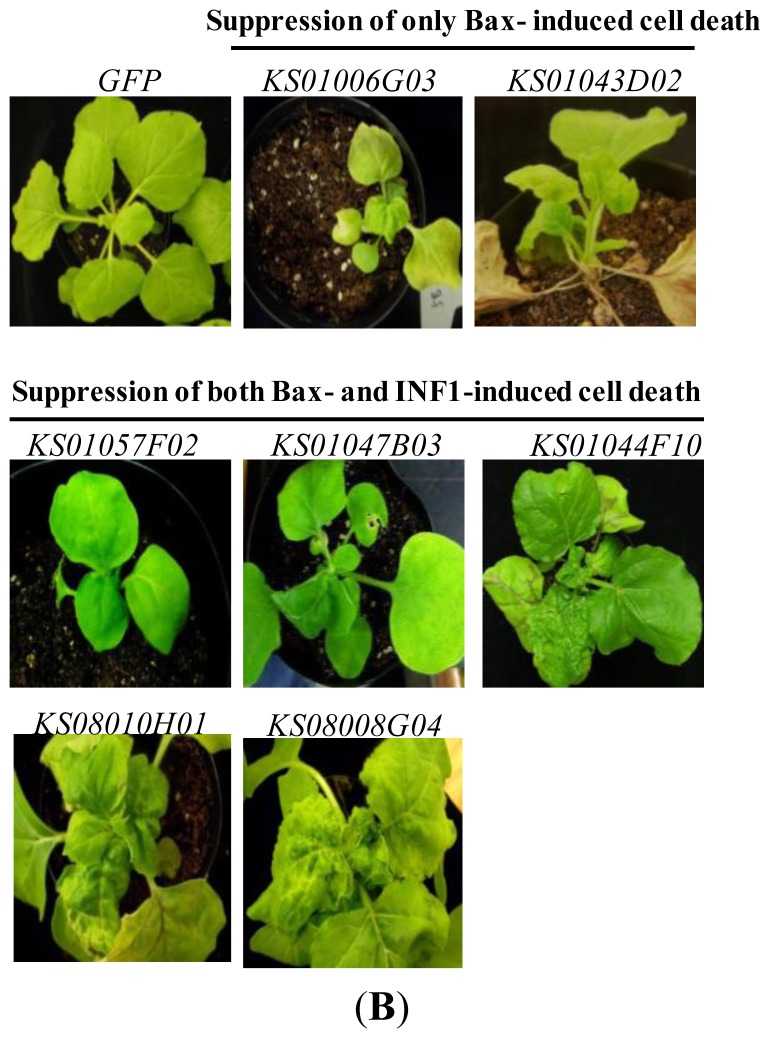
Cell death phenotypes in *N. benthamiana* after transient expression of Bax and INF1 in plants treated with indicated pepper TRV clones. (**A**) *N. benthamiana* plants were infected with 459 *TRV*-defense-associated pepper ESTs or *pTRV-GFP* as the control. After three weeks, the upper leaves of each silenced plant were exposed to Bax or INF1. Images of plants exhibiting suppressed cell death responses were taken three days post-inoculation; and (**B**) Morphological alterations of plants in which Bax and/or INF1-induced cell death-associated pepper genes were silenced. Images were taken three weeks after VIGS was initiated.

To identify genes involved in Bax- or INF1-mediated cell death responses, transient expression of Bax or INF1 was induced in silenced *N. benthamiana* plants, and the plants were scored for the presence of a cell death phenotype. Transient expression of Bax or INF1 in the leaves of *TRV-GFP*-inoculated *N. benthamiana* plants induced cell death within two days (Figure 2A). In an initial screen in which three replicate plants for each pepper *TRV2* derivatives were exposed to Bax or INF1, 78 clones were identified in which Bax- or INF1-induced cell death was compromised. Two further independent biological replicate experiments were performed with these 78 affected clones, from which seven clones were identified that were reproducibly compromised in the cell death response. Five of these clones exhibited reduced cell death when treated with Bax or INF1, and two further clones exhibited reduced cell death only with Bax treatment (Figure 2A, Table 2). We confirmed the VIGS of the corresponding *N. benthamiana* genes in the silenced plants after three weeks of TRV infection by semi-quantitative reverse transcription polymerase chain reaction (RT-PCR) using the specific primers for the each *N. benthamiana* homologs (Supplementary Tables S1 and S2). The expression levels of all seven *N. benthamiana* homologs were significantly reduced in the silenced plants compared with that of in the control plants which were silenced with the pTRV-*GFP* (Supplementary Figure S1). The identified genes, representing 1.5% of the set, encode proteins with putative functions in protein stability and secondary metabolism, as well as unknown functions. This is a relatively low proportion of the gene set when compared to a previous VIGS screen for HR genes [6]. This may be due to the genes used in this study, which were chosen based on their induction during infection with a non-host pathogen, *Xag* 8ra; accordingly, *Xag* 8ra-induced HR might share few genes with Bax- or INF1-induced cell death. The identification of genes involved in both INF1- and Bax-induced cell death and genes only involved in Bax-induced cell death indicates the presence of common and distinct components of these two cell death pathways.

### 2.3. Silencing of the Putative Calcium-Binding Protein CML30-Like and Cys-3-His Zinc Finger Protein Suppressed Bax-Induced Cell Dath, but Not INF1-Induced Cell Death

**Figure 3:**
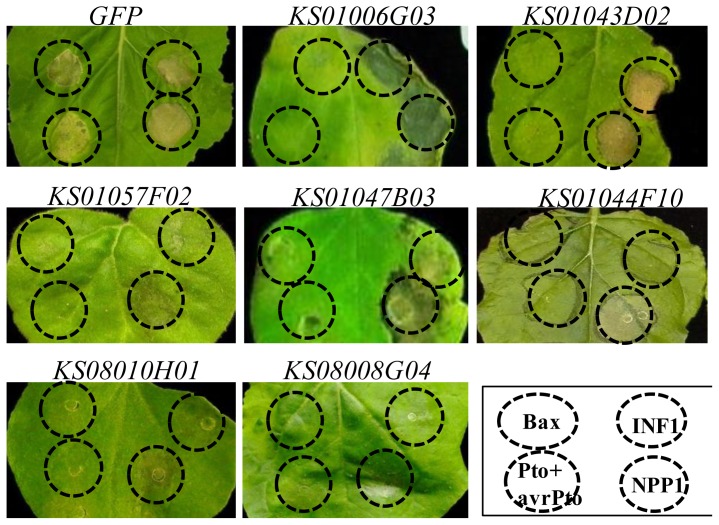
Comparison of cell death induced by Bax, Pto-avrPto, INF1, and NPP1 in plants silenced with *TRV*-pepper ESTs. *N. benthamiana* plants were inoculated with the indicated pepper *TRV* clones. After three weeks, upper leaves were exposed to Bax, Pto-avrPto, INF1, and NPP1, and images were taken three days later.

Two pepper clones, *KS01043D02* and *KS01006G03*, suppressed only Bax-induced cell death (Figure 2A). To investigate whether this suppression was Bax-specific, two further cell death pathways were induced in these two pepper clones, namely, those affected by Pto-avrPto [6] and NPP1 [22]. Pto is an *R* gene, encoding serine/threonine kinase, which confers resistance to *Pseudomonas syringae.* pv. *tomato* by recognizing and interacting with the pathogen type III effector protein AvrPto. NPP1 is necrosis-inducing *Phytophthora* protein 1 [22]. In both *KS01043D02-* and *KS01006G03-*silenced plants, Pto-avrPto-induced cell death was inhibited, similar to Bax-induced cell death; however, NPP1-induced cell death was not inhibited (Figure 3). In *TRV-GFP* control plants, cell death for all treatments was observed at two days post-inoculation.

The putative protein encoded by *KS01006G03* has high homology with a calcium-binding CML30-like protein. The biological function of this protein is unknown; however, previous research indicates that 13 calmodulin genes are differentially expressed in tobacco plants during pathogen-induced HR and wounding, and that calmodulin is one of the major signaling components involved in Tobacco Mosaic Virus-induced cell death in tobacco [23,24]. In the current study, by using VIGS, we showed that a putative calmodulin-like gene is involved in Bax- and Pto-avrPto-induced cell death, but not in INF1- or NPP1-induced cell death in *N. benthamiana*. We also observed that the protein encoded by this gene appears to be involved in development, as VIGS with a fragment of the gene caused growth defects (Figure 2B).

*KS01043D02* encodes a hypothetical protein of unknown function that contains zinc finger and ankyrin-repeat domains. BLAST analysis indicates that putative homologs exist in tomato and *Arabidopsis* (*LOC101258155* and *At2G40140*, respectively). A T-DNA insertion mutant of *AtSZF1* (*At2G40140*) shows increased susceptibility to salt stress [25], indicating that this protein might have roles in biotic and abiotic defense mechanisms, including HR.

### 2.4. Bax- and INF1-Induced Cell Death Were Suppressed in *N. benthamiana* Plants in Which One of Five Pepper Genes Were Silenced

VIGS using five pepper ESTs, namely, *KS01044F10*, *KS01047B03*, *KS01057F02*, *KS08010H01*, and *KS08008G04*, resulted in a noticeable suppression of both Bax- and INF1-induced cell death (Figure 2A). This indicates that the orthologous genes in *N. benthamiana* of these five pepper genes might be essential for cell death induced by Bax and INF1. Interestingly, all these gene- silenced plants exhibited defected morphologies such as stunted growth and abnormal leaves, indicating that the five genes might not only be involved in cell death but, also, in plant growth and development. The five genes encode a putative luminal binding protein (BiP) (*KS01044F10*), an acyl carrier protein (ACP) (*KS01047B03*), a pectate lyase P56-like protein (*KS01057F02*), a mannose-binding lectin (*KS08010H01*), and a 60S ribosomal protein L10 (*KS08008G04*) (Table 2). Cell death suppression in the silenced plants was prolonged; therefore, we assumed that these genes are essential to general cell viability. However, when the five silenced plants were treated with Pto-avrPto and NPP1, they exhibited variable cell death inhibition phenotypes (Figure 3). Silencing of *KS01044F10* and *KS01047B03* effectively compromised Pto-avrPto-mediated HR, but did not suppress NPP1-induced cell death. *KS01057F02-*, *KS08010H01-*, and *KS08008G04*-silenced tobacco plants exhibited a weakened NPP1-induced cell death response as well as suppressed Pto-avrPto-induced cell death (Figure 3).

Luminal binding proteins (BiPs) are key components required for protein maturation and transport, and serve as master regulators of the unfolded protein response (UPR), the signaling pathway triggered by the accumulation of misfolded or unfolded proteins in the mammalian endoplasmic reticulum [26,27]. In plants, application of UPR inducers such as tunicamycin leads to the accumulation of transcripts corresponding to ER chaperones, such as BiP, indicating the possible conservation of UPR in plants [28]. Therefore, it is conceivable that BiP functions in Bax-, Pto-avrPto-, and INF1-mediated cell death via its role in UPR; however, NPP1-induced cell death was not affected by BiP.

ACP regulation has been the focus of several studies of lipid biosynthesis in plants [29]. Plant cells must adjust to changing lipid requirements during development and under varied environmental conditions, and so must control the expression of the many genes that are involved in the fatty acid and lipid synthesis pathways. In mammalian cells, down-regulation of mitochondrial ACP compromises protein lipolyation and the respiratory complex, and eventually results in cell death [30]. This study provides the first evidence that ACP is associated with plant cell death, in particular with Bax-, Pto-avrPto-, and INF1-coupled cell death.

A previous VIGS Pto-AvrPto-induced HR screen identified diverse ribosomal proteins as cell death components [6], and ribosomal proteins are differentially expressed upon elicitation of a defense response in tomato [31]. The HR requires transcriptional up-regulation of various defense-related genes [32], and it may require an increase in ribosomal proteins for the concomitant increase in translational activity.

The *KS08010H01*, mannose-binding lectin (CaMBL1), has been shown previously to play a role in plant cell death and defense responses [33]. Transient overexpression of the *CaMBL1* induced cell death on pepper leaves and transgenic *Arabidopsis* plants expressing the *CaMBL1* increased defense responses to bacterial pathogen invasion [33]. Although, they have not studied a suppression of the HR in the *CaMBL1*-silenced pepper plants, it is tempting to speculate that the mannose-binding lectin we observed here could be involved in the general cell death responses or in the defense responses to fungal pathogen.

The pectate lyase P56-like protein (*KS01057F02*) has been previously shown to be involved in extension of pollen tube. The role of pectate lyase neither in plant innate immunity nor in cell death has been previously investigated. In this study, the observed loss-of-function phenotype suggests that pectate lyase plays an important role as a positive regulator of basal defense such as HR, and also has a role in plant development.

## 3. Experimental Section

### 3.1. Plant Materials and VIGS

*Nicotiana benthamiana* seeds were germinated in soil, and plants were grown in a glasshouse at 23 °C. DNA from 459 pepper EST clones [19], was double-digested with *Eco*RI/*Xho*I and, in a separate reaction, with *Bam*HI/*Kpn*I. The resulting fragments were blunted by T4 DNA polymerase and then ligated into the *pTRV2* silencing vector. *pTRV* RNA1 vector (*pTRV1*) or *pTRV2* derivatives (contained pepper ESTs) were mobilized into *Agrobacterium tumefaciens* strain GV2260 by the freeze-thaw method of transformation [20,21]. *A. tumefaciens* cells carrying *pTRV1* and *pTRV2* derivatives were initially cultured in 5 mL of liquid Luria-Bertani (LB) media containing 50 μg/mL kanamycin and 100 μg/mL rifampicin and grown overnight at 28 °C. *A. tumefaciens* cells were harvested and re-suspended in infiltration media (10 mM MgCl2, 10 mM MES buffer, pH 5.6, 200 μM acetosyringone), adjusted to an absorbance (OD_600_) of 0.8–1.0, and incubated at room temperature for 3 h. Subsequently, the mixtures of *A. tumefaciens* containing *pTRV1* and *pTRV2* derivative plasmids were mixed together in a ratio of 1:1 and infiltrated into the fully expanded leaf of ~three-week-old *N. benthamiana* plants using a 1 mL needleless syringe. VIGS experiment was conducted two times with three biological replications for each construct. VIGS of chili pepper was performed as previously described [21].

### 3.2. Agrobacterium-Mediated Transient Expression

Overnight cultures of *A. tumefaciens* strain GV3101 containing the following plasmids were harvested by centrifugation: 35S-promoter driven *Bax* [11], *Pto* and *avrPto* [6], and *pGR106 INF1* and *NPP1* [15,22] cells were re-suspended in infiltration media (10 mM MgCl2, 10 mM MES buffer, pH 5.6, 200 μM acetosyringone) to an OD_600_ of 0.5–0.1 and incubated for 2 h at room temperature*. A. tumefaciens* were infiltrated into the silenced tobacco leaves two weeks after infection with *pTRV* plasmids. Experiment was conducted three times with six replications for each experiment.

### 3.3. Non-Host Pathogen-Induced Pepper ESTs

Hot chili pepper EST candidate genes were categorized into functional groups. Gene sequences were identified from the pepper consensus sequence [http://genepool.kribb.re.kr/new/index.php-Solanaceae Gene Indices (six species)] and these sequences were used to perform BLASTN and BLASTX analysis using the GO (http://www.geneontology.org/) and TAIR (http://www.Arabidopsis.org) databases.

### 3.4. RT-PCR Analysis

Total RNA was extracted from leaf tissues of the silenced plants using the TRI reagent according to the manufacturer’s instructions (MRC, Cincinnati, OH, USA). Total extracted RNA was treated with 1 unit of RNase-free DNase (Promega, Madison, WI, USA) for 10 min at 37 °C, and purified using the TRI reagent. The first-strand cDNA was synthesized using 1 μg of DNase-treated RNA, oligo d(T) primer, and Moloney murine leukemia virus reverse transcriptase(M-MLV RT, Invitrogen, Rockville, MD, USA) following standard protocols and Semi-quantitative PCR was performed in a total volume of 20 μL in AccuPower PCR PreMix (Bioneer, Daejeon, Korea). To ensure that only host genes and not the viral RNA transcripts were amplified, the RT transcriptase reactions were performed using oligo d(T) primers. The forward primer F1 and reverse primer R2 were used to detect endogenous gene transcripts. The primers used in RT-PCR analysis were shown in the Supplementary Table S2.

## 4. Conclusions

In this study, we transiently overexpressed cell death induction components in a population of *N. benthamiana* plants, in which various candidate HR genes from pepper were silenced using VIGS. Using this strategy, we identified seven pepper ESTs that are involved in INF1- and/or Bax-induced cell death responses. Further studies of these seven candidate genes are required to determine whether they are also required for other defense signaling pathways, and at which stages the proteins act in the signal transduction cascades leading to HR.

## Supplementary Information



## Figures and Tables

**Table 1:** Pepper expressed sequence tags (ESTs) showing morphological change in VIGSed *N. benthamiana* plants.

EST ID	BLASTX *	VIGSed phenotypes
	**Catalytic activity/ligase activity**	
KS01017F01	Q8RU74_LYCES Dehydroquinate synthase (EC 4.6.1.3)	Stunted
KS26035G04	Q5Z8K3_ORYSA Putative ZEITLUPE	Stunted

	**Catalytic activity/oxidoreductase activity**	
KS01002H05	Q6S5A3_TROMA Sterol delta-7 reductase	Dead
KS13009F01	Q9SVA9_ARATH Cytochrome P450-like protein	Leaf necrotic spot

	**Catalytic activity/transferase activity**	
KS13055B07	Q5DKV0_TOBAC Adenosine kinase isoform 1T	Wrinkled leaf

	**Catalytic activity/hydrolase activity**	
KS23052B07	chitinase/lysozyme [*Nicotiana tabacum*]	Pale green
KS08010H01	probable pectate lyase P56-like [*S. lycopersicum*]	Stunted & wrinkled leaf
KS01052C01	Q8H272_LYCES Metacaspase 1 (Fragment)	Wrinkled leaf

	**Binding/ion binding**	
KS12078F02	calmodulin [*Capsicum annuum*]	Stunted
KS01006G03	probable calcium-binding protein CML30-like [*S. lycopersicum*]	Stunted

	**Binding/protein binding**	
KS01047E07	O04133_SOYBN SRC2	Stunted

	**Binding/nucleic acid binding**	
KS01060G11	Q93XV7_9ROSI Hydroxypyruvate reductase (EC 1.1.1.29)	Small leaf size
KS12047A04	Q9XI07_ARATH F8K7.13 protein (Putative transcriptional regulatory protein)	Weak dwarf

	**Binding/others**	
KS01044F10	BIP5_TOBAC Luminal binding protein 5 precursor (BiP 5)	Stunted
KS01057F02	mannose-binding lectin [*C. annuum*]	Stunted
KS14050G06	Q41424_SOLTU Chlorophyll a/b binding protein	Pale green

	**Structural molecule activity**	
KS10105B11	Q9SCM3_ARATH 40S ribosomal protein S2 homolog (At3g57490)	Stunted & curling leaf
KS08008G04	60S ribosomal protein L10 [*S. melongena*]	Stunted & curling leaf

	**Transporter activity**	
KS24014C12	Q93Y42_ARATH Coatomer delta subunit (Delta-coat protein)	Dead
KS01047B03	Q9XF20_CAPCH Acyl carrier protein	Stunted
KS08017G06	O80774_ARATH Hypothetical protein At2g34250	Stunted & curling leaf

	**Transcription regulator activity**	
KS01043D02	Q9XEE6_ARATH Hypothetical Cys-3-His zinc finger	Stunted
KS09041E04	Q710C3_SPIOL Sigma factor (Fragment)	Pale green
KS09088A04	Q7Y039_LYCES MADS-box protein 5	Abnormal leaf
KS12065G09	Q8RXK4_ARATH Hypothetical protein At4g38900	Stunted

	**Unknown**	
KS20002B10	later embryo abundant protein [*Prunus dulcis*]	Stunted
KS01047F02	Q9C9T7_ARATH Hypothetical protein F25P22.17	Stunted
KS01056A09	No_hits	Complete dead
KS20014C01	No_hits	Leaf local dead
KS07002E11	AAD25142.1 (Hypothetical protein At3g24506)	Pale green
KS07012E06	Q9FJZ7_ARATH Arabidopsis thaliana genomic DNA, chromosome 5,	Chlorosis
KS19065G03	Q9C9V9_ARATH Putative golgi transport complex protein; 67058-70172	Stunted & curling leaf

* Blast hit at http://genepool.kribb.re.kr/new/index.php-Solanaceae Gene Indices (six species) [19].

**Table 2:** Pepper ESTs involved in Bax- or INF1-mediated cell death responses.

EST ID	BLASTX *	*E*-value	Phenotypes		

			Morphology	HR	

				Bax	INF
KS01044F10	glucose-regulated protein 78 (BiP), partial [*S. lycopersicum*]	2.0 × 10^−87^	stunted	X	X
KS01047B03	Acyl carrier protein [*C. chinense*]	3.0 × 10^−58^	stunted	X	X
KS08008G04	60S ribosomal protein L10 [*S. melongena*]	7.0 × 10^−121^	stunted & wrinkled leaf	X	X
KS08010H01	probable pectate lyase P56-like [*S. lycopersicum*]	8.0 × 10^−77^	stunted & wrinkled leaf	X	X
KS01057F02	mannose-binding lectin [*C. annuum*]	1.0 × 10^−77^	stunted	X	X
KS01006G03	probable calcium-binding protein CML30-like [*S. lycopersicum*]	1.0 × 10^−77^	stunted	X	Δ
KS01043D02	Hypothetical Cys-3-His zinc finger protein [*A. thaliana*]	1.0 × 10^−125^	stunted	X	O

* BLAST hit at http://genepool.kribb.re.kr/new/index.php-Solanaceae Gene Indices (six species) [19]. Morphological phenotypes were observed three weeks after VIGS was performed. Suppression phenotypes varied: X: cell death was suppressed three days post-inoculation (dpi); Δ: cell death was delayed 3 dpi; O: cell death was the same as that of the GFP control plants.
