# Supplementary material for: Identification of Novel Pepper Genes Involved in Bax- or INF1-Mediated Cell Death Responses by High-Throughput Virus-Induced Gene Silencing
Source: Int J Mol Sci. 2013 Nov 19;14(11):22782–95. doi: 10.3390/ijms141122782 (PMC3856090; doi:10.3390/ijms141122782)
Supplement: Supplementary file 1 [file ijms-14-22782-s001.pdf]

Supplementary Information

Table S1. Putative pepper EST homologs identified in *Nicotiana benthamiana*.

Pepper EST ID	*N. benthamiana* homolog *	*E*-value	Annotation
KS01044F10	lcl|Nbv3K585732136	0.00E+00	Luminal-binding protein 5 (BiP 5), Precursor
KS01047B03	lcl|Nbv3K745624312	4.00E-75	Acyl carrier protein 4, chloroplastic (ACP), Precursor
KS08008G04	lcl|Nbv3K645786840	2.00E-47	60S ribosomal protein L10
KS08010H01	lcl|Nbv3K725838694	1.00E-53	Pectate lyase, Precursor
KS01057F02	lcl|Nbv3K725838062	5.00E-31	Epidermis-specific secreted glycoprotein EP1, Precursor
KS01006G03	lcl|Nbv3K585739567	4.00E-43	Probable calcium-binding protein CML45
KS01043D02	lcl|Nbv3K645789478	1.00E-159	Zinc finger CCCH domain-containing protein 29 (AtC3H29)

* BLAST hit at http://benth-web-pro-1.ucc.usyd.edu.au/blast/blast.php.

Table S2. Primer sequences for semi-quantitative RT-PCR.

Primers	Sequences (5' to 3')
lcl|Nbv3K585732136 F	*CCACTTACTCGGGCTCGTTT*
lcl|Nbv3K585732136 R	*AGGGTTGACACCCTTGTTGG*
lcl|Nbv3K745624312 F	*ACTTGACCCCGTGTCACTTG*
lcl|Nbv3K745624312 R	*GCTTCCTCAAGTCCCATGACA*
lcl|Nbv3K645786840 F	*GTGCTCGTGTTGCAATTGGT*
lcl|Nbv3K645786840 R	*GATCTTTTGTCGGCCAGGGA*
lcl|Nbv3K725838694 F	*TATCGATGCTGTCGCTGCTT*
lcl|Nbv3K725838694 R	*TCCACTGCTTCCGCCAATAG*
lcl|Nbv3K725838062 F	*TATCATGCGCTGGGTATGGG*
lcl|Nbv3K725838062 R	*GGGTGTCGGTGGGATAATCG*
lcl|Nbv3K585739567 F	*CTCCACCTCTTCCCTGCATA*
lcl|Nbv3K585739567 R	*ACAAAAATGGCGGCTCCTAGT*
lcl|Nbv3K645789478 F	*CTTTTGCAAAGCAGCGGAGT*
lcl|Nbv3K645789478 R	*TGTCCTCGCTATTGAAGCCC*
NbActin F	*TGGACTCTGGTGATGGTGTC*
NbActin R	*CCTCCAATCCAAACACTGTA*

Figure S1. Suppression of homologous gene expression in silenced *N. benthamiana* plants. Transcript levels of each gene were analyzed by RT-PCR. Total RNAs were extracted from leaf tissues of GFP-control or gene-silenced plants at 24 h after Bax inoculation. RNA (1 µg) was used in cDNA synthesis and PCR was performed using the primers described in the Supplemental Table S2. PCR products were sampled from each PCR cycle number indicated at the top and were separated on an agarose gel and stained with ethidium bromide.

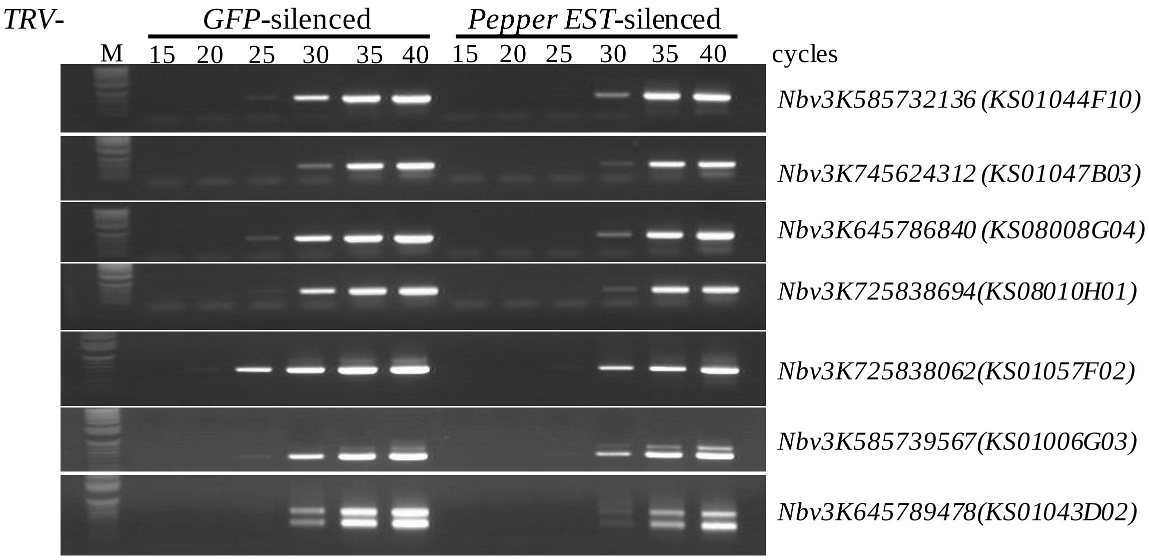

© 2013 by the authors; licensee MDPI, Basel, Switzerland. This article is an open access article distributed under the terms and conditions of the Creative Commons Attribution license (http://creativecommons.org/licenses/by/3.0/).
